# TB transmission is associated with prolonged stay in a low socio-economic, high burdened TB and HIV community in Cape Town, South Africa

**DOI:** 10.1186/s12879-020-4828-z

**Published:** 2020-02-10

**Authors:** Rebecca Tadokera, Linda-Gail Bekker, Barry N. Kreiswirth, Barun Mathema, Keren Middelkoop

**Affiliations:** 1 Desmond Tutu HIV Centre, Institute of Infectious Disease and Molecular Medicine, University of Cape Town, PO Box 13801, Mowbray Observatory, Cape Town, 7705 South Africa; 2 NRF/DST Centre of Excellence for Biomedical Tuberculosis Research, Medical Research Council Centre for Tuberculosis Research, Stellenbosch University, Cape Town, South Africa; 3 Department of Medicine, University of Cape Town, Cape Town, South Africa; 4 Public Health Research Institute Tuberculosis Center, New Jersey Medical School, Rutgers University, Newark, NJ USA; 5 Department of Epidemiology, Mailman School of Public Health, Columbia University, New York City, NY USA

**Keywords:** Tuberculosis, TB transmission, Clustering, Socio-economic status, RFLP, Molecular epidemiology

## Abstract

**Background:**

While several studies have assessed the associations between biological factors and tuberculosis (TB) transmission, our understanding of the associations between TB transmission and social and economic factors remains incomplete. We aimed to explore associations between community TB transmission and socio-economic factors within a high TB-HIV burdened setting.

**Methods:**

We conducted a cross-sectional molecular epidemiology study among adult patients attending a routine TB clinic. Demographic and clinical data were extracted from TB registers and clinical folders; social and economic data were collected using interviewer-administered questionnaires; *Mycobacterium tuberculosis* isolates were genotyped and classified as clustered/non-clustered using *IS*6110-based Restriction Fragment Length Polymorphism. Composite “social” and “economic” scores were generated from social and economic data. Data were analyzed using StataCorp version 15.0 software. Stratified, bivariable analyses were performed using chi-squared. Wilcoxon signed rank tests; univariable and multivariable logistic regression models were developed to explore associations in the social, economic, traditional and composite TB risk factors with TB transmission.

**Results:**

Of the 505 patient *Mtb*  strains, 348(69%) cases were classified as clustered and 157(31%) were non-clustered. Clustered cases were more likely to have lived longer in the study community, (odds ratio [OR] = 1.05, 95% Confidence interval [C.I]:1.02–1.09, *p* = 0.006); in the same house (OR = 1.04, C.I: 0.99–1.08, *p* = 0.06); and had increased household crowding conditions (i.e fewer rooms used for sleeping, OR = 0.45, C.I:0.21–0.95, *p* = 0.04). Although a higher proportion of clustered cases had a low economic score, no statistically significant association was found between clustering and either the economic score (*p* = 0.13) or social score (*p* = 0.26).

**Conclusions:**

We report a novel association between *Mtb* transmission and prolonged stay within a high burdened community. Transmission was also associated with fewer rooms for sleeping in a household. Increased social interaction and prolonged residence in a high burdened community are important factors linked to *Mtb* transmission, possibly due to increased probability of higher effective contact rates. The possible importance of degrees of poverty within low socio-economic setting warrants further study.

## Background

While significant progress has been made to halt and reverse tuberculosis (TB) cases and deaths globally, the burden of TB remains enormous, with the World Health Organization (WHO) reporting an estimated 10 million incident cases every year [1]. Huge challenges still remain in the fight against TB, particularly in the Low-Medium-income countries (LMIC) [1, 2]. With TB incidence rates of over 781/100000, and 60% of incident TB cases co-infected with HIV-infected, South Africa remains one of the world’s top six high TB and HIV burdened countries [1]. Molecular epidemiological studies have reported that much of the burden of TB disease in South Africa is due to ongoing transmission [3, 4]. Traditional TB molecular epidemiology studies have sought to distinguish between disease due to recent *Mycobacterium tuberculosis* (*Mtb)* infection or transmission compared to reactivation of latent infection [5–7]. TB cases with identical strains clustered for a given time and place are often considered to be part of a common transmission chain [3, 8]. Thus, clustering is often used as a proxy for recent transmission [2, 9, 10]. Studies from various settings have reported varying findings on risk factors for clustering such as age, immigrant status, HIV infection homelessness, alcoholism, intravenous drug use, social mixing and treatment failure [11–15]. There are discrepancies in the importance of these factors across studies, particularly between the high [16] and low income country contexts [17, 18]. There remains a need to further explore and understand the factors driving *Mtb* transmission in poor socio-economic communities with a high burden of both TB and HIV. The identification of such risk factors could inform targeted control measures and interventions aimed at interrupting TB disease transmission chains and reducing TB incidence, in line with the WHO’s End TB Strategy [19]. In this study, we aimed to investigate how social, economic and composite factors related to community TB transmission (clustering vs. non-clustering) in a high TB and HIV burdened community setting.

## Methods

We conducted a post hoc analysis of data from a cross-sectional study among TB cases resident in a peri-urban township in Cape Town, South Africa from 2006 to 2010. This community had a population of 13,180 people in 2006 which grew to 16,851 in 2010. Approximately 1 in every 4 adults in this community was HIV-infected as of 2008 [3, 20]. In the same year TB case notifications were as high as 2000/100000, despite the presence of a functional primary care TB facility and increasing antiretroviral therapy (ART) coverage [21]. High rates of TB transmission have previously being reported in this community [22].

Eligible TB clients attending the community TB clinic were identified and informed about the study. Inclusion criteria were TB disease notified from 2006 to end 2010, residency in the study community, and a willingness to provide written informed consent. Clinical and demographic data were extracted from the TB registers and clinical folders. TB and socio-economic data were collected using interviewer-administered questionnaires that were translated to the participant’s local language. The questionnaires captured data on TB history, TB contacts, sexual history, and socio-economic such as occupation, income level, educational level and living conditions.

HIV testing and counseling (and referral for treatment, where required) was conducted according to the national HIV guidelines [23]. Sputum specimens were obtained from TB suspects in accordance with the national TB testing, diagnostic and treatment guidelines [24]. Mycobacteriological tests, including microscopy and culture, were performed on the sputum specimens as described elsewhere [25].

*Mtb* isolates from participants were analysed using *IS*6110-based Restriction Fragment Length Polymorphism (RFLP), [26] performed at the Public Health Research Institute (PHRI), Tuberculosis Centre Laboratory, New Jersey. Based on the genotyping data, strains were classified using standard software and tools [27]. Previous analysis of the *Mtb* strains showed that the dominant strain families in the study population were the W-Beijing (29% of participants) and CC-related strains (24%) [28].

### Definitions

A strain was defined as a genetic variant of an isolate [29]. A unique strain was an isolate with an RFLP pattern that occurred in only one participant within the study dataset and was designated as a non-clustered strain. A cluster was defined as > 1 specific strain detected in different individuals within the study population. Strains from dually infected participants were analyzed as individual samples (*n* = 2). Retreatment TB cases resulting from the same strain as the patient’s previous TB episode were presumed to be due to relapse and were excluded from analysis. Strains with < 6 copies of IS6110 (low bandwidth strains) are known to be poorly differentiated by the RFLP technique and so were excluded from further analysis [29].

Composite scores were generated for economic and social risk factors. Variables for inclusion in the composite scores were decided prior to analysis but finalized based on assessment for collinearity. Education level, employment status, income level, electricity access, having a toilet in the house, and number of rooms used for sleeping (a surrogate for house size) were all classified as economic factors and comprised the composite economic score out of 11. The type of house was strongly correlated with electricity supply to the house (variance inflation factor [VIF]: 9.8) and was therefore not included in the composite score. Each variable was assigned a value ranging from 0 to 4 (depending on the number of categories in the variable), with a higher score corresponding to higher economic status. For example, education was scored 0 for no formal education and 4 if a participant had tertiary education; a score of 0 was given if there was no electricity in the participant’s house and 1 if the house had electricity. The following factors were incorporated in the social score with a maximum score of 9: alcohol consumption in past 12 months, shebeen (informal tavern) patronage in past 12 months, meeting regularly with a group, regular use of a minibus taxi, number of new sexual partners within the past 6 months, number of houses on the residential plot and number of occupants living in the same house. It is also notable that while the majority of those participants who reported visiting shebeens also consumed alcohol, there was a proportion that visit shebeens for social or other reasons besides alcohol consumption. Furthermore, not all alcohol consumption occurs on shebeen premises. Given the weak collinearity between alcohol drinking and shebeen patronage (VIF: 2.2) we chose to keep both these variables in the social score. Each variable was assigned a value of 0, 1 or 2 (depending on the number of categories in the variable), with a higher score corresponding to greater social interaction. Both the economic and social scores were divided into binary variables at the median (to generate a “low” and “high” economic and social score).

Additional relevant risk factors not classifiable as social or economic risk factors included in the analyses were: a history of TB contacts, recent death in family, tobacco smoking, period of residence in the same house and in the community, history of mine work, history of imprisonment and time spent outside study community.

Our analysis was restricted to adult participants (≥15 years of age) who had both socio-economic questionnaire data and an RFLP-based *Mtb* genotype available. We excluded children (*n* = 12) on the presumption that social and economic behaviors of children were different from those of adults.

### Statistical analysis

Data were analysed using Stata 15.0 (StataCorp, College Station, Texas). Bivariable analyses were performed using chi-squared and Wilcoxon signed rank tests to explore baseline differences in the socio-economic and traditional TB risk factors between the clustered and the non-clustered participants, as appropriate. Univariable logistic regression models were used to calculate odds ratios and associations between stratified risk factors (such as income categories) and clustered and non-clustered participants. Multivariable logistic regression models were developed to determine associations between TB transmission (clustering), social and economic score and the other specified risk factors. Variance inflation factors were calculated to assess for collinearity between risk factors in multivariable regression models.

## Results

### Study population

**Fig. 1:**
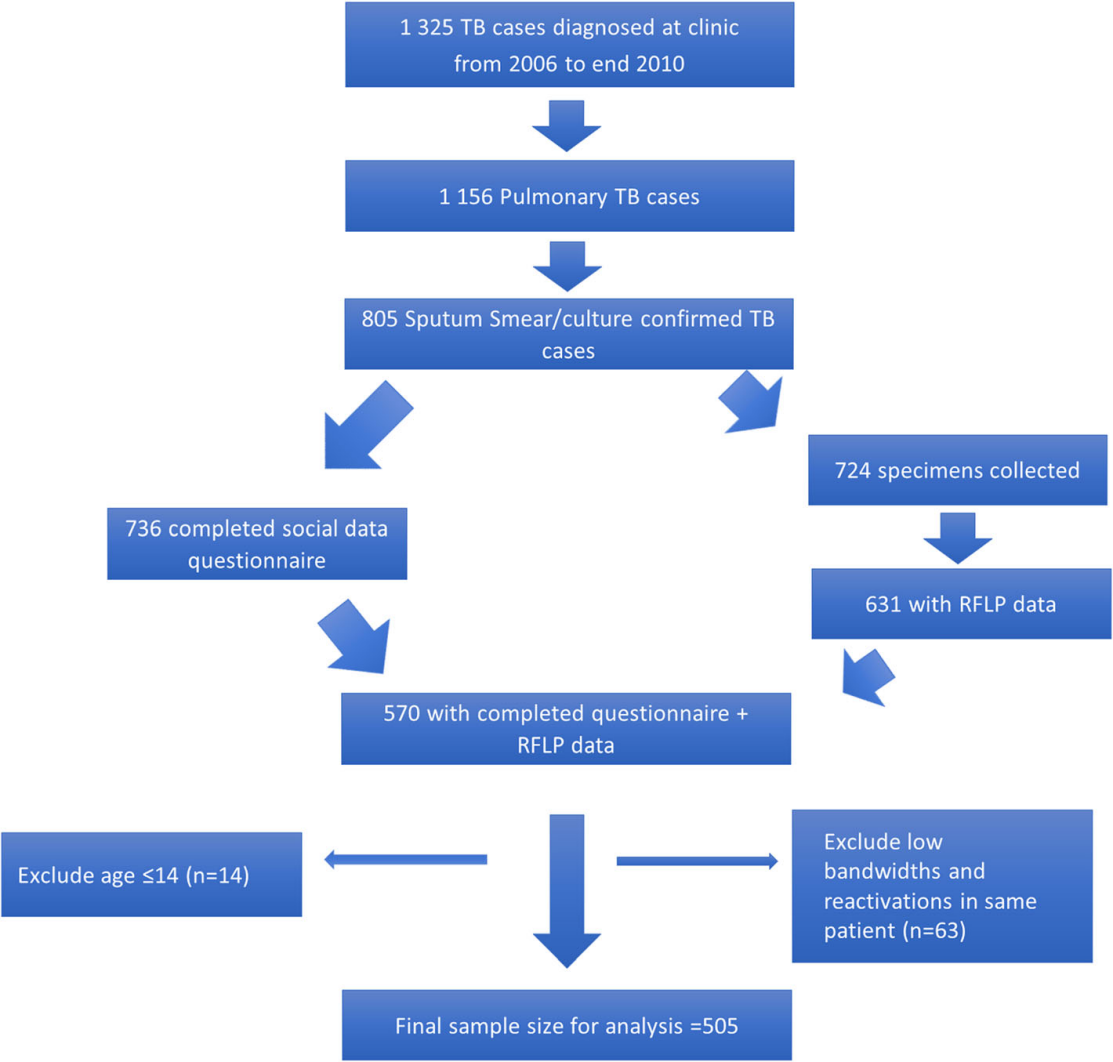
Consort diagram of the recruitment and sample selection process

Figure 1 is a consort diagram summarizing the study sample selection. In summary, out of the 1325 TB cases in the study period, there were 805 sputum positive TB cases. Six hundred thirty-one participants had RFLP data while 736 had socio-economic (questionnaire) data available. All in all, 570 had both socio-economic and RFLP data available. Following additional exclusions as described above, the final sample comprised of 503 participants, and 505 TB strains (2 of the participants had dual infection). There are no significant differences between patients included in this analysis and the broader community TB cohort, by age (*p* = 0.25), gender (*p* = 0.07), HIV status (*p* = 0.31) or new vs retreatment TB (*p* = 0.90).

Of the 505 strains, 348 (69%) were classified as clustered while the remaining 157 (31%) were classified as non-clustered. There was an even distribution in the baseline demographic factors when comparing the clustered and non-clustered cases. Age ranged from 16 to 77 years and was uniformly distributed across the two groups (*p* = 0.68) as was gender (43% vs 40% females; *p* = 0.53). The majority of the study participants (93%) were isiXhosa speaking.

### Economic and social risk factors

No significant differences were noted in most of the economic variables between clustered and non-clustered cases (Table 1). Overall 29% vs 30% of the participants in non-clustered versus clustered groups reported having acquired only primary level education respectively, while 16 and 18% in the respective groups had secondary and/or tertiary education. At 67%, unemployment levels were high in this study population, with low monthly household income reported: only 2% of households earned more than R5000/ month.

**Table 1:** Summary of bivariable analysis of possible socio-economic TB transmission risk factors, *n* = 505

Characteristic	Total: *N* = 505	Non-clustered *N* = 157 (31%)	Clustered *N* = 348 (69%)	*P*-value	OR (95% CI)
Education, n(%)					
No formal education	23 (5)	8 (5)	15 (4)		1.00 (reference)
Some primary school	149 (29)	45 (29)	104 (30)	0.66	1.23 (0.49; 3.11)
Some high school	248 (49)	75 (48)	173 (50)	0.65	1.23 (0.50; 3.03)
Matric	81 (16)	27 (17)	54 (15)	0.90	1.07 (0.40; 2.83)
Tertiary education	4 (1)	2 (1)	2 (1)	0.57	0.53 (0.06;4.53)
Employed, n (%)	163 (32)	53 (34)	110 (32)	0.63	0.91 (0.61;1.35)
Income ZAR, median (IQR)	1600 (1060;2250)			0.82*	1.0 (0.99;1.00)
< 2000	95 (61)	30 (58)	65 (62)		1.00 (reference)
R2000-R5000	57 (37)	19 (36)	38 (37)	0.82	0.92 (0.46;1.86)
> 5000	4 (2)	3 (6)	1 (1)	0.11	0.15 (0.02;1.54)
Housing Conditions, n (%)					
Informal housing, n (%)	449 (89%)	138 (88)	311 (89)	0.63	0.86 (0.48;1.56)
Electricity in house, n (%)	486 (96)	150 (96)	336 (96)	0.58	1.31 (0.50;3.38)
Toilet in house, n (%)	47 (9)	13 (8)	34 (10)	0.59	1.20 (0.61;2.34)
Number of occupants in house, median (IQR); n (%)	3 (2;4)				
1–2	214 (42)	65 (41)	149 (42)		1.00 (reference)
3–4	166 (33)	50 (32)	116 (33)	0.96	1.01 (0.65;1.57)
5 or more	125 (25)	42 (27)	83 (24)	0.54	0.86 (0.54;1.38)
No. of rooms used for sleeping, median (IQR)	1 (1; 2)				
1 room	299 (59)	89 (57)	210 (60)		1.00 (reference)
2 rooms	175 (35)	53 (34)	122 (35)	0.91	0.98 (0.65; 1.47)
3–6 rooms	31 (6)	15 (9)	16 (5)	0.04	0.45 (0.21; 0.95)
Consumed alcohol in last 12 months, n (%)	185 (37)	49 (31)	136 (39)	0.09	1. 41 (0.94; 2.11)
Been to a shebeen in the last12 months, n (%)	113 (22)	29 (18)	84 (24)	0.16	1.40(0.88; 2.25)
Regularly takes a taxi, n (%)	478 (95)	146 (93)	332 (93)	0.27	1.56 (071; 3.45)
Meet group regularly, n (%)	44 (9)	15 (10)	29 (8)	0.65	0.86 (0.45; 1.66)
Number of new sex partners in the last 6 months, n (%):					
0	399 (79)	117 (75)	282 (81)		1.00 (reference)
1–3	106 (21)	40 (25)	66 (19)	0.10	0.68 (0.44; 1.07)

Summary of the bivariable analysis of possible socio-economic TB transmission risk factors by clustered vs. non-clustered cases

*continuous variable; Wilcoxon signed rank test

Living conditions were similar across both non-clustered and clustered study groups. The majority of study participants lived in informal dwellings (89%). Only 9% of the participants reported having a toilet in the house with the remainder using a communal water tap for household water supply (*p* = 0.59 for comparison across groups). However, 96% did report having access to electricity in their house. The number of occupants living in a household ranged between 2 and 17 persons, with a median of 3 occupants per house. The majority of households (59%) reported having a single room for sleeping. Non-clustered cases were more likely to have more rooms for sleeping (9% had ≥3 rooms compared to 5% of the clustered cases; *p* = 0.04). Although not statistically significant, clustered cases were more likely to report alcohol consumption (39% vs. 31% *p* = 0.09), and patronage of a shebeen in the past 12 months (24% vs. 18%, *p* = 0.16). A large proportion of the study participants reported using a taxi regularly for transport in both groups (93% of both clustered and non-clustered cases).

The median composite economic score was 4 out of 11 (interquartile range [IQR]: 3–5), (Table 2). Overall non-clustered cases trended towards higher economic scores, but this was not statistically significant (32% over the median vs 25% in clustered cases; *p* = 0.13). The median composite social score was 4 out of 9 (IQR: 3–5). There was no statistical difference between the social scores for clustered vs non- clustered cases (28 vs 33% respectively; *p* = 0.26).

**Table 2:** Summary of economic and social composite risk scores between clustered and no-clustered cases

Characteristic	Total: *N* = 505	Non-clustered *N* = 157 (31%)	Clustered *N* = 348 (69%)	*P*-value	OR (95% CI)
Economic score (as per methods)					
(Continuous), median (IQR)	4 (3; 5)	4 (3; 5)	4 (3; 5)	0.20	0.91 (0.79; 1.05)
High economic score (≥5), n (%)	138 (27)	50 (32)	88 (25)	0.13	0.72 (0.48; 1.11)
Social Score					
(Continuous), median (IQR)	4 (3; 5)	4 (3; 5)	4 (3; 5)	0.71	1.02 (0.90; 1.17)
High Social Score (≥5), n (%)	159 (531)	44 (28)	115 (33)	0.26	1.28 (0.84; 1.92)

Summary of the analysis of associations between the associations economic and social composite risk scores vs clustered/von-clustered cases

### Other possible TB risk factors at time of diagnosis

In the 477 (94%) study participants who had a known HIV status, proportions of HIV positive participants were similar between clustered and non-clustered cases (63 vs. 68%, *p* = 0.29; Table 3). Furthermore, there was no differences reported between study groups in knowing a TB patient. A non-statistically significant greater proportion of clustered cases reported a recent death (of any cause) in their household (16% vs.10%, *p* = 0.11) with 24% of clustered cases reporting a household member known to have died recently due to TB, compared to 25% of non-clustered cases (*p* = 0.94). No statistical associations were found in the traditional risk factors of smoking and recent time in prison. As strong association was identified between clustered cases and increasing duration of time (in years) living in the study community (median of 6 years versus 4 in non-clustered cases; *p* = 0.004) and a trend towards a similar association with time lived in the same house (median = 3 years vs. 2 years respectively, *p* = 0.06) (Table 3).

**Table 3:** Summary of bivariable associations between clustering and other possible TB transmission risk factors

Characteristic	Total: n (%)	Non-clustered cases, n (%)	Clustered cases n (%)	*P*-value	OR (95% CI)
	*N* = 505	*n* = 157	*n* = 348		
Known HIV Status	477 (94)	148 (94)	329 (95)	0.90	1.05 (0.47; 2.38)
HIV positive	316 (66)	93 (63)	223 (68)	0.29	1.24 (0.83;1.87)
Know anyone currently being treated for TB	153 (30)	47 (30)	106 (30)	0.91	1.03 (0.68;1.55)
Know anyone ever treated for TB	93 (18)	29 (18)	64 (18)	0.98	0.99 (0.61; 1.62)
Had a death in the house in past 2 years	70 (14)	16 (10)	54 (16)	0.11	1.62 (0.89; 2.93)
Been to clinic or hospital in the last 6 months	293 (58)	94 (60)	199 (57)	0.57	0.901 (0.61; 1.31)
Smoked tobacco in last 6 months	172 (34)	47 (30)	125 (36)	0.19	1.31 (0.87; 1.97)
Been in prison in the last 6 months	12 (2)	3 (2)	9 (3)	0.65	1.36 (0.36;5,10)
Time lived in current house (years), median (IQR)	3.0 (0.8; 8.0)	2 (0.6; 6)	3 (0.9; 8)	0.06	1.04 (0.99; 1.08)
Time lived in study community (years), median (IQR)	6.0 (3; 11)	4 (1; 9)	6 (3; 11)	0.004	1.05 (1.02; 1.09)

Summary of other possible TB risk factors at time of diagnosis that could not be classified as social or economic

### Multivariable analysis between TB transmission and socio-economic risk factors

For the multivariable analysis, we explored the association between TB transmission and selected risk factors (Table 4). Variables assessed in the regression model included those variables with a trend towards association with clustering in the bivariable analysis (*p* < 0.2), including knowing someone who had died in the past 2 years, being a smoker and time spent outside the study community. Time lived in current house strongly correlated to time lived in the study community and so was not included in the regression model (*r* = 0.78). Based on our prior knowledge about the risk factors for TB transmission, age, gender and HIV status are potential confounding variables, and so were adjusted for in the regression model. Table 4 shows a summary of the multivariable analysis, based on these variables. There was a positive association observed between a longer duration of stay in the study community and clustering (OR = 1.05, C.I: 1.01 to 1.09). However, no other statistical association were identified. The model did not change substantively when HIV status was excluded (42 cases did not have a known HIV status).

**Table 4:** Summary of multivariable analysis between TB transmission and selected socio-economic risk factors (*n* = 477 cases^a^)

Characteristic	Odds ratio	95% Confidence Interval	*P*-Value
Age	0.99	0.96; 1.01	0.18
Gender	1.11	0.71; 1.75	0.65
HIV Status	1.32	0.87; 2.02	0.20
Time resident in community (years)	1.05	1.01; 1.09	0.02
Knowing someone who died of TB (last 2 years)	1.60	0.86; 3.00	0.14
Smoked	1.22	0.76;1.95	0.42
Social Score (binary)	1.12	0.72; 1.74	0.62
Economic score (binary)	0.71	0.46–1.12	0.14

Summary of multivariable analysis between TB transmission and selected socio-economic risk factors

^a^42 cases did not have known HIV status

## Discussion

The role of socio-economic factors in TB transmission remains a pertinent question in many high burden communities. In this study, based in a high TB burden community of generally low socio-economic status, we explored associations between socio-economic risk factors and *Mtb* strain clustering. Prolonged stay within this community was strongly associated TB transmission. Despite the high degree of homogeneity in the demographic characteristics of the study population at baseline, a higher proportion of clustered vs non-clustered cases had lower economic scores, although this was not statistically significant.

We analyzed economic risk factors for transmission, both individually and by creating a composite economic score. We observed a significant negative association between TB transmission and the number of household rooms used for sleeping in this study. Participants who reported having more than 3 rooms for sleeping were less likely to be part of a transmission cluster. This association may point to less close indoor contact time, particularly for lengthy overnight periods, hence a reduced risk of TB transmission for those who have more spacious or less crowded houses. Moreover, a trend towards individuals with lower income being more likely to be part of a TB transmission cluster was also noted. The number of participants earning salaries in the higher income category (>R5000; [±$350] per month) was very small and this may have reduced our power to show a statistically significant association, and further investigation of this finding is warranted. Taken individually, the remaining economic factors did not yield any strong statistical associations with TB transmission. Lower composite economic scores were noted in a higher proportion of clustered cases, although this was not statistically significant. Our findings are in agreement with other researchers who have reported that poor socio-economic conditions may predispose to TB transmission [15, 30, 31]. But further, given the setting of a low economic community, these findings may hint at the possibility of a “sliding-scale effect of poverty” even in such communities, with individuals at the lower end of the economic scale being at potentially greater risk for acquiring TB infection. The factors that are linked to economic status, which in turn may explain this association are complex and may include poor nutritional status, poor living conditions and health status among other related and potential underlying factors [10]. The questionnaire administered in this study did not enable us to explore these complexities in detail, which may in some part explain the lack of statistical associations. Our findings are in general agreement with other studies which have reported a socio-economic gradient between countries, within countries and even within communities [12, 30].

In order to quantify social interaction and its possible associations with TB transmission, we created a composite social score. We found no overall association between TB transmission and the composite social score. However, we identified other individual-level factors associated with transmission. Specifically, both a longer stay in the same house and longer duration of living in the community were associated with belonging to a TB transmission cluster. These associations may be a measure of prolonged and persistent exposure to *Mtb* in a community with a high burden of TB disease, with a higher effective contact rate and thus an increasing chance of acquiring TB infection for participants living in the community for longer periods of time. Although an intuitive finding, to our knowledge this is the first study to show that prolonged stay within a high burden TB community with high rates of ongoing TB transmission [22] results in an increased risk of being part of a TB transmission cluster. A weak association was also noted between belonging to a transmission cluster and individuals who reported alcohol consumption in the past year and although we did not quantify alcohol consumption, there are plausible biological as well as social rationales for this finding.

While our results identified potential epidemiological links between TB transmission and socio-economic risk factors, we were surprised by the paucity of associations with many of the risk factors investigated, and with the composite social and economic scores. However, a study by Mathema et al. in South African gold-miners also could not establish any risk factors for TB transmission and this finding was posseted to be due to a universally high risk for disease in that population [32]. Our findings in this study point to a similar scenario, with difficulty identifying specific transmission risk factors in a generally low socio-economic community with exceptionally high TB disease and transmission rates [33, 34]. Some historical studies have reported the role of crowding and poor living conditions on the risk of TB transmission within households, and Andrews et al. have further suggested that targeted interventions among the poor may be one of the most effective interventions to reduce TB transmission [35]; an approach that would be supported by our findings in this study.

While the inference of recent transmission of tuberculosis from clustered strains has a number of recognized limitations [29] our interpretation is strengthened by supporting evidence of high Mtb transmission rates in the community [22], the notable diversity of circulating strains [28], the study duration and the discriminatory power of RFLP [29]. Potential limitations for our study include information potential biases due to missing data. Firstly, participation in the study was voluntary; although recruitment was excellent with over 90% of eligible patients enrolled in the questionnaire component of the study. Secondly, we were not able to obtain genotyping data for all enrolled patients. We have previously reported few significant differences in patients with RFLP data and those without [28]: of note multi-drug resistant TB (MDR-TB) patients were more likely to have RFLP data and patients who had died were less likely to have RFLP data. However, there was no statically significant difference between those patients with and without RFLP data in terms of age, gender, new versus retreatment TB or HIV or ART status [28]. Missing specimen genotype data as well as the recognized limitations to the discriminatory power of RFLP [29] may also have resulted in misclassification of apparent unique strains, with an underestimation of clustering. Another potential limitation in this study is that our sample size of 505 strains may have lacked power to detect small statistical differences. This could potentially explain the non-statistically significant trends for some of the risk factors analyzed in this study. In addition, the socio-economic combined scores used have not been validated. Further work to confirm these findings in larger populations across different populations could bring more definitive insights into the social and economic factors linked with TB transmission that would guide national policy guidelines in high burdened settings.

## Conclusions

In summary, our study found that prolonged residence in a high burdened community and less crowding in households are important factors linked to TB transmission. The association between TB transmission and prolonged stay in a high transmission community although intuitive, has not been demonstrated in previous studies. While the association between degrees of poverty and TB transmission was not conclusive, further studies using more comprehensive questionnaires and a larger sample size into this question are warranted.

## Data Availability

The datasets that were used for this manuscript are available from the corresponding author upon reasonable request.

## Abbreviations

aOR: Adjusted odds ratio
ART: Antiretroviral therapy
CI: Confidence interval
HIV: Human immuno-virus
IS: Insertion sequence
LMIC: Low/medium income countries
*Mtb*: *Mycobacterium tuberculosis*
OR: Odds ratio
RFLP: Restriction Fragment Length polymorphism
TB: Tuberculosis
WHO: World Health Organization
ZAR: South African Rands
